# Diagnostic accuracy of the Finnish Diabetes Risk Score (FINDRISC) for undiagnosed T2DM in Peruvian population

**DOI:** 10.1016/j.pcd.2018.07.015

**Published:** 2018-12

**Authors:** Antonio Bernabe-Ortiz, Pablo Perel, Juan Jaime Miranda, Liam Smeeth

**Affiliations:** a CRONICAS Center of Excellence in Chronic Diseases, Universidad Peruana Cayetano Heredia, Lima 18, Peru; b Faculty of Epidemiology and Population Health, London School of Hygiene and Tropical Medicine, London WC1E 7HT, UK; c Department of Medicine, School of Medicine, Universidad Peruana Cayetano Heredia, Lima 31, Peru

**Keywords:** Diabetes mellitus, type 2, Glucose tolerance test, Risk assessment, Screening, Diagnostic test

## Abstract

**Aims:**

To assess the diagnostic accuracy of the Finnish Diabetes Risk Score (FINDRISC) for undiagnosed T2DM and to compare its performance with the Latin-American FINDRISC (LA-FINDRISC) and the Peruvian Risk Score.

**Materials and methods:**

A population-based study was conducted. T2DM and undiagnosed T2DM were defined using oral glucose tolerance test (OGTT). Risk scores assessed were FINDRISC, LA-FINDRISC and Peruvian Risk Score. Diagnostic accuracy of risk scores was estimated using the c-statistic and the area under the ROC curve (aROC). A simplified version of FINDRISC was also derived.

**Results:**

Data from 1609 individuals, mean age 48.2 (SD: 10.6), 810 (50.3%) women, were collected. A total of 176 (11.0%; 95%CI: 9.4%–12.5%) were classified as having T2DM, and 71 (4.7%; 95%CI: 3.7%–5.8%) were classified as having undiagnosed T2DM. Diagnostic accuracy of the FINDRISC (aROC = 0.69), LA-FINDRISC (aROC = 0.68), and Peruvian Risk Score (aROC = 0.64) was similar (p = 0.15). The simplified FINDRISC, with 4 variables, had a slightly better performance (aROC = 0.71) than the other scores.

**Conclusion:**

The performance of FINDRISC, LA-FINDRISC and Peruvian Risk Score for undiagnosed T2DM was similar. A simplified FINDRISC can perform as well or better for undiagnosed T2DM. The FINDRISC may be useful to detect cases of undiagnosed T2DM in resource-constrained settings.

## Highlights

• Diagnostic accuracy of FINDRISC, LA-FINDRISC and Peruvian Risk Score was similar.
• A simplified version of FINDRISC was created with only four variables.
• The performance of the FINDRISC in Peruvian population was moderate.
• Forty percent of individuals with T2DM are unaware of diagnosis.

## 1 Introduction

Globally, there is an increase in the burden of type 2 diabetes mellitus (T2DM): the age-standardized prevalence of T2DM has increased from 4.3% to 9.0% among men and from 5.0% to 7.9% among women in the last four decades [1]. T2DM is also responsible for about 2 million deaths every year worldwide [2], [3] and USD 825 billion are estimated to be spent in T2DM-related healthcare [1], [4].

Identifying individuals with undiagnosed T2DM can be an important approach to prevent or delay T2DM complications [5], though, universal screening for T2DM at the population level is still controversial [6]. Thus, although, the American Diabetes Association recommends T2DM testing for all adults starting at age 45 years regardless of weight, or those who are overweight or obese and have one or more additional risk factor for T2DM [7]; the Disease Control Priorities group recommends testing individuals at high-risk of T2DM (i.e. those aged ≥40 years, individuals with family history of T2DM, obesity, physical inactivity, or dyslipidemia) [6].

The identification of T2DM cases can be better addressed using a two-step approach. Thus, in the first step, a risk score – defined as “an objective assessment of the probability of the presence or future development of an adverse health condition” [8] – can be applied to identify subjects at high risk of having or developing T2DM, and, in the second step, a confirmatory test (fasting glucose, oral glucose tolerance test [OGTT] or glycated hemoglobin [HbA1c]) can be performed, but only among those categorized as high risk in the previous step [9].

Different risk models, also known as risk scores, have been developed to detect T2DM cases. Some of them are useful to detect undiagnosed (prevalent) T2DM cases, whereas other ones predict the development of new (incident) T2DM cases [10]. The Finnish Diabetes Risk Score (FINDRISC) is a questionnaire to identify individuals at high risk of developing T2DM, and was created using a prospective cohort of individuals aged between 35 and 64 years [11]. Original questions included age, body mass index, waist circumference, physical activity, daily consumption of fruits, berries or vegetables, history of anti-hypertensive drug treatment, and history of high blood glucose [12]. However, later studies added family history of T2DM to the model and modified diet patterns and physical activity questions. Despite being widely used for estimating the risk of developing T2DM within the following ten years, the FINDRISC has been also evaluated as a tool to identify undiagnosed T2DM, abnormal glucose tolerance and metabolic syndrome [13], [14], [15]. Although this score is widely used in many Latin America settings [16], [17] and even a Latin America FINDRISC (LA-FINDRISC) has been described [18], its diagnostic accuracy needs to be assessed in other resource-constrained settings. Therefore, this study aimed to assess the diagnostic accuracy of the FINDRISC for undiagnosed T2DM. In addition, we compared the performance of the FINDRISC, the LA-FINDRISC and the Peruvian Risk Score.

## 2 Materials and methods

### 2.1 Source of data

Analyses were conducted using data from a population-based cross-sectional study carried out in Tumbes, a semiurban area in the north of Peru. Based on projections of the 2007 national census, Tumbes has 243,000 inhabitants in an area of 4670 km^2^ [19]. The rationale for selecting this setting was because prevalence of obesity, by body mass index (32% vs. 18%), and T2DM, by fasting plasma glucose (10% vs. 7%), is over the national average [20].

### 2.2 Participants

Eligible participants were those aged between 30 and 69 years, full time resident in the study area (i.e. ≥6 months) and able to understand procedures and provide informed consent. Women that reported being pregnant or individuals having any physical disability preventing anthropometric measurements (weight, height, blood pressure or waist circumference) or those bedridden were excluded from the study.

A sex-stratified, single-stage random sampling strategy was conducted using the most updated census available in the study area (2014). To avoid potential clustering of behavioral factors, only one participant per household was invited to participate in the study.

### 2.3 Outcome

T2DM was the outcome variable of interest, and was defined according to the World Health Organization threshold using the OGTT [7]. Individuals who were not aware of having T2DM diagnosis and had fasting glucose level ≥126 mg/dL (≥7.0 mmol/L) or 2-h plasma glucose ≥200 mg/dL (≥11.1 mmol/L) were classified as undiagnosed T2DM.

### 2.4 Predictors

Socio-demographic, behavioral and anthropometric variables included in the manuscript were those related to the risk models evaluated: FINDRISC [13], LA-FINDRISC [18], and the Peruvian Risk Score [21]. These variables were: age (in years), body mass index (in kg/m^2^), waist circumference (in cm), physical activity (at least 30 min per day), daily consumption of fruits and vegetables (at least one portion per day), history of anti-hypertensive drug treatment (yes vs. no), history of high blood glucose (whether participant has ever been found to have high blood glucose in a health examination, during an illness or during pregnancy: yes vs. no), and family history of T2DM (score according to relatives with T2DM diagnosis). Information about scoring of these risk models is available in Appendix A (Table A1, Table A2).

### 2.5 Procedures

After informed consent, participant’s data was collected using tablets and measurements were obtained by well-trained staff. Participants responded to a face-to-face questionnaire. An application built using Open Data Kit (ODK) was utilized using tablets. Using the application, we obtained data about factors potentially associated with T2DM, including sociodemographic, behavioral variables, personal medical history, and familial medical history focused mainly on glucose metabolism disorder. Specific questions of the risk models were also included.

After completing questionnaires, measurements of standing height were carried out using a stadiometer and standardized procedures. Weight was assessed using a bio-electrical impedance device (TBF-300A, TANITA Corporation, Tokyo, Japan), as well as waist circumference was assessed in triplicate using standard techniques. Heart rate, systolic and diastolic blood pressure were also evaluated in triplicate using an automatic monitor OMRON HEM-780 (OMRON Healthcare, Illinois, US), previously validated for adult population.

Trained laboratory staff explained procedures for blood sample collection. Participants were asked to provide venous blood sample for oral glucose tolerance test (OGTT) after a minimum of 8 and a maximum of 12 h of fasting. First blood sampling was obtained at the first moment of the appointment, after verifying fasting period was accomplished. A total of 7.5 ml of venous blood sample was drawn to assess fasting glucose. After that, a load of 75 g of anhydrous glucose in a volume of 300 ml was used as recommended [7]. Two hours after, a new blood sample was obtained to measure glucose levels. In the mid-time, questionnaires and clinical measurements were performed. Blood testing was carried out by a certified Peruvian laboratory located in Lima. Laboratory staff was blinded to results of questionnaires and measurements. Glucose was measured in serum using a Cobas Modular Platform automated analyzer and reagents supplied by Roche Diagnostics. Quality control for glucose measurements had <1 for the coefficient of variation, a reference range provided by Bio-Rad, an independent assessment company (www.biorad.com).

### 2.6 Statistical analyses methods

Analysis was performed using STATA 13.0 for Windows (StataCorp, College Station, TX, US). Initially, characteristics of study population were tabulated using proportions in the case of categorical variables, and mean and standard deviation (SD) for continuous variables. After overall participants’ description, all cases of known T2DM were further excluded from analyses. Then, the prevalence and 95% confidence interval (95% CI) of undiagnosed T2DM was estimated.

Scoring of FINDRISC, LA-FINDRISC and Peruvian Risk Score was determined using original coefficients. Then, diagnostic accuracy of these scores was estimated using the c-statistic and graphically with the area under the ROC curve. Optimal empirical cut-off following the method suggested by Youden was estimated [22], and sensitivity and specificity were reported. Comparison between diagnostic accuracy of risk scores was conducted using the *roccomp* command in STATA.

Finally, the FINDRISC was simplified by including only variables independently associated with undiagnosed T2DM in our sample using backward elimination strategy in logistic regression. The risk factors in the simplified model were each assigned a weighted score (i.e. by dividing the regression coefficients in the final model by the lower coefficient and then rounding them up to the nearest integers as in a previous report) [23]. Diagnostic accuracy of the simplified FINDRISC was also assessed using area under the ROC curve as well as sensitivity and specificity.

### 2.7 Ethics

The protocol, informed consent and questionnaires were approved by Ethical Institutional Committees at the Universidad Peruana Cayetano Heredia, Lima, Peru, and London School of Hygiene and Tropical Medicine, London, UK. This work has been carried out in accordance with the Declaration of Helsinki.

## 3 Results

### 3.1 Characteristics of the study population

A total of 2114 individuals were invited to participate in the study; 486 (22.9%) rejected participation and 16 (0.8%) women were pregnant and further excluded. Of the 1612 (76.3%) participants enrolled in the study, three did not complete all blood procedures; and therefore, only 1609 were further analyzed. Overall, the mean age of the analyzed sample was 48.2 (SD: 10.6) and 810 (50.3%) were women. About a third of the population had <7 years of education, and two thirds were currently working. Detailed characteristics of the study population are shown in Table 1.

**Table 1:** Characteristics of the study population: comparison between total population and those with OGTT results.

		Total population	With OGTT results
		N = 1609	N = 1504
		N (%)	N (%)
Sex	Female	810 (50.3%)	750 (49.9%)
Age	Mean (SD)	48.2 (10.6)	47.6 (10.6)
Education level	<7 years	519 (32.3%)	466 (31.0%)
	7–11 years	749 (46.6%)	708 (47.1%)
	12+ years	341 (21.2%)	330 (21.9%)

Socioeconomic status (tertiles)	Lowest	540 (33.6%)	497 (33.1%)
	Middle	550 (34.2%)	517 (34.4%)
	Highest	519 (32.3%)	490 (32.6%)

Currently working	Yes	1091 (67.8%)	1035 (68.8%)
Health insurance	Yes	1469 (91.3%)	1368 (91.0%)
T2DM in first degree-relatives	Yes	539 (33.5%)	468 (31.1%)
Daily smoking	Yes	92 (5.7%)	86 (5.7%)
Alcohol disorder	Yes	121 (7.5%)	121 (8.1%)
Physically active (≥30 min/day)	Yes	1098 (68.2%)	1036 (68.9%)
Fruits and vegetables intake	At least one/day	841 (52.3%)	789 (52.5%)
Body mass index (kg/m^2^)	Mean (SD)	28.0 (4.6)	28.0 (4.7)
Obesity by BMI	BMI ≥30 kg/m^2^	476 (29.6%)	450 (29.9%)
Waist circumference (cm)	Mean (SD)	93.7 (10.4)	93.6 (10.4)
Obesity by WC	Based on IDF	1277 (79.4%)	1186 (78.9%)
Systolic blood pressure (mmHg)	Mean (SD)	119.9 (16.7)	119.5 (16.3)
Diastolic blood pressure (mmHg)	Mean (SD)	79.7 (10.4)	79.5 (10.3)
Blood pressure treatment	Yes	128 (8.0%)	106 (7.1%)
Hypertension status	Yes	417 (25.9%)	370 (24.6%)
Self-reported high glucose	Yes	159 (9.9%)	56 (3.7%)

### 3.2 Prevalence of T2DM and undiagnosed T2DM

A total of 176 (11.0%; 95% CI: 9.4%–12.5%) out of 1609 participants were classified as having T2DM. One hundred five (6.5%; 95% CI: 5.4%–7.8%) individuals were aware of T2DM diagnosis and were excluded from further analysis. Based on the OGTT results, 71 (4.7%; 95% CI: 3.7%–5.8%) were classified as having undiagnosed T2DM. Of the 71 who met criteria for T2DM on OGTT, 56 (78.9%) met diagnostic criteria based on fasting glucose alone. Characteristic of those with OGTT results (n = 1504) were similar to those of the total study population (n = 1609), except in the case of self-reported history of high glucose levels (Table 1).

### 3.3 Diagnostic accuracy of risk score models for undiagnosed T2DM

The mean score of the FINDRISC was 8.9 (SD: 4.2, range: 0–24) points, whereas results for the LA-FINDRISC and the Peruvian Risk Score were 8.6 (SD: 4.4, range: 0–24) and 1.5 (SD: 1.1, range: 0–4), respectively.

When assessing the diagnostic accuracy of the FINDRISC, the area under the ROC curve was 0.69 (95% CI: 0.64–0.74), with an empirical optimal cut-off of 11, and a sensitivity of 69%; whereas the area under the ROC curve for the LA-FINDRISC was 0.68 (95% CI: 0.63–0.74), with a cut-off of 10, and a sensitivity of 70.4%. When assessing the diagnostic accuracy of the Peruvian Risk Score, the area under the ROC curve was 0.64 (95% CI: 0.58–0.70), with an empirical cut-off of 2, and a sensitivity of 64.8% (See details in Table 2). There were no differences in the diagnostic accuracy of the aforementioned risk scores (p = 0.15).

**Table 2:** Diagnostic accuracy of risk score models for undiagnosed T2DM.

	FINDRISC	LA-FINDRISC	Peruvian Risk Score	Simplified FINDRISC
	Estimate (95% CI)	Estimate (95% CI)	Estimate (95% CI)	Estimate (95% CI)
Area under the ROC curve	0.69 (0.64–0.74)	0.68 (0.63–0.74)	0.64 (0.58–0.70)	0.71 (0.66–0.76)
Empirical cut-off	11	10	2	3
Sensitivity	69.0% (56.9%–79.5%)	70.4% (58.4%–80.7%)	64.8% (52.5%–75.8%)	85.9% (75.6%–93.0%)
Specificity	66.8% (64.3%–69.2%)	59.1% (56.5%–61.7%)	53.7% (51.0%–56.3%)	46.7% (44.1%–49.3%)
Positive predictive value	9.4% (7.0%–12.2%)	7.9% (5.9%–10.2%)	6.4% (4.8%–8.6%)	7.4% (5.7%–9.4%)
Negative predictive value	97.8% (96.6%–98.6%)	97.6% (96.3%–98.5%)	96.8% (95.4%–97.9%)	98.5% (97.3%–99.3%)
Likelihood ratio positive	2.1 (1.8–2.5)	1.7 (1.5–2.0)	1.4 (1.2–1.7)	1.6 (1.5–1.8)
Likelihood ratio negative	0.5 (0.3–0.7)	0.5 (0.4–0.7)	0.7 (0.5–0.9)	0.3 (0.2–0.5)
Diagnostic odd ratio	4.5 (2.7–7.5)	3.4 (2.1–5.8)	2.1 (1.3 –3.5)	5.3 (2.8–10.4)

### 3.4 Adaptation and simplification of FINDRISC for Peruvian population

**Fig. 1:**
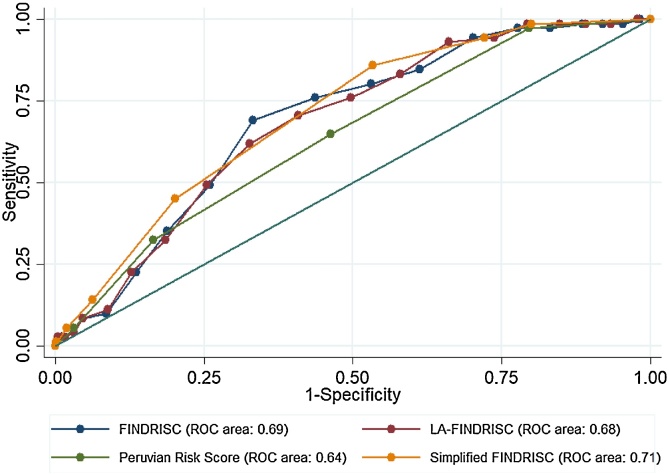
Comparison of area under the ROC curves using the FINDRISC, LA-FINDRISC, the Peruvian Risk Score and the simplified risk scores.

When simplifying FINDRISC, variables independently associated with undiagnosed T2DM were: waist circumference (p = 0.008), blood pressure treatment (p = 0.004), history of high blood glucose (p = 0.005), and family history of T2DM (p = 0.01). Coefficients and scores are detailed in Table 3. The area under the ROC curve of the simplified FINDRISC was 0.71 (95% CI: 0.66–0.76), and with an empirical cut-off ≥3, the sensitivity and specificity were 85.9% and 46.7%, respectively. Thus, the diagnostic accuracy of the simplified FINDRISC score was similar to the FINDRISC (p = 0.24) and LA-FINDRISC (p = 0.19), but superior than the Peruvian Risk Score (p = 0.02, Fig. 1)

**Table 3:** Beta coefficients of the simplified FINDRISC for undiagnosed T2DM in Peruvian population.

	Bivariable model		Final model^a^		Score
	Coef. (SE)	OR (95% CI)	Coef. (SE)	OR (95% CI)	
Age (vs. <45 years)					
≥45 and <55 years	0.39 (0.29)	1.48 (0.84–2.62)			
≥55 and <65 years	0.26 (0.33)	1.29 (0.68–2.44)			
≥65 years	0.34 (0.50)	1.40 (0.52–3.74)			

Body mass index (vs.<25 kg/m^2^)					
≥25 and <30 kg/m^2^	0.46 (0.36)	1.58 (0.78–3.21)			
≥30 kg/m^2^	0.99 (0.36)	2.70 (1.34–5.43)			

Waist circumference (vs. F < 80 cm/M < 94 cm)					
F: ≥80 and <88 cm/M: ≥94 and <102 cm	1.10 (0.44)	3.02 (1.26–7.21)	1.04 (0.45)	2.82 (1.17–6.76)	2 (vs. 0)
F: ≥88 cm/M: ≥102 cm	1.46 (0.41)	4.31 (1.92–9.65)	1.30 (0.42)	3.65 (1.62–8.26)	3 (vs. 0)

Physical activity (vs. no)					
At least 30 min per day	0.13 (0.26)	1.14 (0.69–1.89)			

Fruits and vegetables intake (vs. no)					
At least once per day	−0.04 (0.24)	0.96 (0.59–1.54)			

Blood pressure medication (vs. no)					
Yes	1.17 (0.33)	3.22 (1.71–6.10)	0.98 (0.33)	2.65 (1.38–5.12)	2 (vs. 0)

History of high blood glucose levels (vs. no)					
Yes	1.32 (0.40)	3.74 (1.70–8.25)	1.19 (0.42)	3.28 (1.44–7.47)	2 (vs. 0)

Family history of T2DM (vs. no)					
Parent, brother, sister or own child	0.63 (0.25)	1.87 (1.16–3.03)	0.61 (0.25)	1.84 (1.13–3.00)	1 (vs. 0)

a The final model was created by backward elimination, keeping only variables significantly associated with undiagnosed T2DM.

## 4 Discussion

### 4.1 Main findings

Our findings demonstrated that the diagnostic accuracy of the FINDRISC, LA-FINDRISC and Peruvian Risk Score for undiagnosed T2DM was similar. However, a simplified version of the FINDRISC, with only four variables, can perform similar to the FINDRISC and LA-FINDRISC, but better than the Peruvian Risk Score. These four variables (i.e. waist circumference, self-report of blood pressure treatment, history of high blood glucose, and family history of type 2 diabetes mellitus) are easy to obtain in clinical practice and thus, could be implemented for detecting undiagnosed T2DM at the population level. In addition, the prevalence of T2DM in the study population was relatively high (11% compared to the national Peruvian average of 7%).

### 4.2 Comparison with previous studies

Worldwide, there are many risk scores created for detecting cases of undiagnosed T2DM, though many of them are for Caucasian [23], [24], [25] and Asian populations [26], [27], [28]. The FINDRISC is a well-known risk score created initially for incident T2DM cases, but currently can be used for T2DM screening [13]. However, previous experience has established that a risk score needs to be adapted, validated, or calibrated in the population where this is planned to be applied as prevalence and distribution of outcomes and risk factors are not similar between settings [29].

The FINDRISC had a moderate performance for T2DM screening in Peruvian population. Our results were similar to previous studies in Latin America [17], [30] and other Spanish-speaking populations like the study of Salinero-Fort et al. in Madrid [31], although the diagnostic accuracy was lower than in Asian [15] or European [13], [32] populations. Moreover, according to our logistic regression modeling, the original FINDRISC can be simplified to only four variables to slightly improve the diagnostic accuracy. A previous report has highlighted the need of a Latin-American version of the FINDRISC (LA-FINDRISC) with changes in the cut-offs of waist circumference [18]; however, our analyses confirm no difference between the FINDRISC and LA-FINDRISC in Peruvian population, and hence, the original score should be kept.

### 4.3 Prevalence of undiagnosed T2DM

This can be the first study estimating the prevalence of T2DM and undiagnosed T2DM using OGTT in Peru. To our knowledge, there are only two previous studies using nationally representative samples to estimate T2DM prevalence, but using fasting glucose: one conducted in 2004–2005 reporting a prevalence of 5% [33], and the other one carried out in 2010–2012 with an estimate of 7% [34]. This number, however, increased up to 10% in Tumbes, in the north of Peru [20], setting where this study was conducted. Using OGTT, our results show that the prevalence of T2DM in Tumbes is 11%, value much greater than the national estimate of 7% [34]. Thus, our results are alarming as we are witnessing the increasing burden of T2DM in resource-constrained settings in a very short period.

Regarding undiagnosed T2DM, our estimates show that about 60% of individuals with T2DM are aware of their disease. Using data of the PERU MIGRANT Study [35], overall T2DM diagnosis awareness was 71%, yet estimates ranged from 0% in rural settings to 74% in urban areas [36]. On the other hand, results using the baseline of the CRONICAS Cohort Study [20] showed that, among all T2DM cases, 61.3% were aware of their diagnosis. In addition, our results are compatible with current reports (range: 24% to 62%) [4]. However, although there are effective interventions to control T2DM [37], [38], unawareness imposes a large economic burden on individuals and families as well as health systems, mainly in resource-constrained settings.

### 4.4 Public Health Relevance

The implementation of the FINDRISC in our population could be useful to detect T2DM cases. According to calculations based on a hypothetic sample of 1000 participants (Table 4), the FINDRISC would detect 76 cases in 371 classified at high risk of T2DM, and for instance, only 37.1% of the 1000 individuals would require a confirmatory test. On the other hand, using the simplified FINDIRSC, with higher sensitivity, a total of 94 cases would be detected, but 568 would be classified as at high risk of T2DM, and for instance, 53% more people would need a confirmatory test, with the consequent increment of the resources and costs.

**Table 4:** Diagnostic accuracy and implications of using a risk score.

Risk score	Sensitivity	Specificity	At high risk of T2DM	T2DM cases detected	Subjects without T2DM
FINDRISC	69.0%	66.8%	371 (37.1%)	76	595
LA-FINDRISC	70.4%	59.1%	441 (44.1%)	77	526
Peruvian Risk Score	64.8%	53.7%	483 (48.3%)	71	478
Simplified FINDRISC	85.9%	46.7%	568 (56.8%)	94	416

All the estimates were calculated assuming that 1000 individuals were screened and a prevalence of 11% of T2DM.

The advantage of the FINDRISC lies on its self-report nature (6 items are easy questions) and the presence of two anthropometrical measurements (body mass index and waist circumference). Our simplified version of the FINDRISC contains only three self-reported items and waist circumference, an anthropometric marker that is easy to measure, making this score implementable in clinical practice. The simplified version of the FINDRISC included waist circumference instead of body mass index as the first one provides a better indicator of accumulation of visceral fat and glucose metabolism deregulation [39]. As one of the barriers to the uptake of risk scores by health practitioners includes the lack of practicality of using the scores and their components [40], an easy measure as the waist circumference might minimize this compared to the use of stadiometer and scale for height and weight of the BMI.

In 2016, the Peruvian Ministry of Health published the Guide of Clinical Practice for Diagnosis, Treatment and Control of Type 2 Diabetes Mellitus in Primary Care. In that guideline, there is no recommendation about the use of risk scores for T2DM screening, but, recommends using fasting plasma glucose among adults between 40 and 70 years with overweight or obesity [41]. The FINDRISC appears then as a very good alternative to screen individuals, especially in areas (semiurban and rural settings) where fasting glucose or other blood markers are not available. A recent systematic review has pointed out the benefit of a two-step approach for T2DM screening, but no study was found in Latin American region [9]. Therefore, there is need to estimate the cost related of using a two-step approach for detecting cases of undiagnosed T2DM in our context.

### 4.5 Strengths and limitations

This study benefits from the use of OGTT to diagnose T2DM. Although data comes from a small region in Peru, the sample was representative from the study area and, for instance, results are inferable. However, this study has also some limitations. First, selection bias might arise as the population sample only included participants aged from 30 to 69 years from a small region in Peru, and thus, our findings can be limited to that group. Thus, further scrutiny is needed to appropriately validate proposed tools at the national level. Second, some desirability and recall bias might be present as some questions show results much higher than expected. For example, more than two thirds participants reported being physically active (i.e. exercise for ≥30 min per day) and almost half of them reported consuming fruits and vegetables at least once a day. Third, power of the study might be an issue as some variables associated with T2DM were not significant in our model. However, the four variables independently associated with undiagnosed T2DM have been associated also in other risk scores in Latin America [21], [30], [42]. In addition, the simplified version of the FINDRISC has been developed with the data of this study and for instance further external validation and evaluation is required. Finally, as in the original FINDRISC, our model was created on the idea of risk stratification instead of individualization [43]; therefore, variables were categorized instead of being kept as numerical in the risk score. However, our idea was to conserve a simple score for detecting cases of undiagnosed T2DM.

## 5 Conclusions

The diagnostic accuracy of the FINDRISC, LA-FINDRISC and Peruvian Risk Score for undiagnosed T2DM was similar. A simplified FINDRISC, with only four variables, can perform similar or better than aforementioned scores. The FINDRISC or its simplified version may be useful to detect cases of undiagnosed T2DM in resource-constrained settings.

## Funding

Antonio Bernabe-Ortiz (grant number: 103994/Z/14/Z) and Liam Smeeth (grant number: 098504/Z/12/Z) are supported by Wellcome Trust, London, UK (www.wellcome.ac.uk).

## Conflict of interest

The authors state that they have no conflict of interest.

## Figures and Tables

**Table A1:** Scoring of FINDRISC and LA-FINDRISC for undiagnosed T2DM.

	FINDRISC	LA-FINDRISC
Age:		
< 45 years	0 points	0 points
45–54 years	2 points	2 points
55–64 years	3 points	3 points
65+ years	4 points	4 points

Body mass index:		
<25 kg/m^2^	0 points	0 points
Between 25 and < 30 kg/m^2^	1 point	1 point
≥30 kg/m^2^	3 points	3 points

Waist circumference:		
Men: <94 cm; women: <80 cm	0 points	0 points
Men: 94–102 cm; women: 80–88 cm	3 points	4 points
Men: >102 cm; women: >88 cm	4 points	

Physical activity (at least 30 min/day):		
Yes	0 points	0 points
No	2 points	2 points
Fruits and vegetables intake:		
Every day	0 points	0 points
Not every day	1 point	1 point

Regular medication for hypertension:		
No	0 points	0 points
Yes	2 points	2 points

History of high glucose levels:		
No	0 points	0 points
Yes	5 points	5 points

Diabetes in relatives:		
No	0 points	0 points
Yes, grandparents, cousins, uncle, aunt	3 points	3 points
Yes, parents, siblings, son, daughter	5 points	5 points

The difference between FINDRISC and LA-FINDRISC is based on score on waist circumference.

**Table A2:** Scoring of the Peruvian Risk Score for undiagnosed T2DM.

	Peruvian Risk Score
Age:	
<55 years	0 points
55+ years	1 point

Waist circumference:	
<90 cm	0 points
90 to <100 cm	1 point
100+ cm	2 points

Diabetes in first-degree relatives	
No	0 points
Yes	1 point
